# Surgical Oncology in 2025: Challenges, Innovations, and the Road Ahead for Young Surgical Oncologists

**DOI:** 10.3390/curroncol32090478

**Published:** 2025-08-25

**Authors:** Jörg Kleeff, Artur Rebelo

**Affiliations:** Department of Visceral, Vascular and Endocrine Surgery, University Hospital Halle (Saale), Martin-Luther-University Halle-Wittenberg, Ernst-Grube-Str. 40, 06-120 Halle (Saale), Germany; artur.rebelo@uk-halle.de

As cancer care becomes increasingly complex and multidisciplinary, the role of surgical oncology continues to evolve at the forefront of innovation. Today, surgical oncologists are not only performing technically demanding procedures but also shaping treatment algorithms, contributing to translational research, and driving improvements in patient outcomes through collaboration with medical and radiation oncology. This expanding scope underscores the importance of a strong, evidence-based foundation to guide clinical decision-making and foster continuous progress in the field.

In this context, journals such as *Current Oncology* are vital platforms for the dissemination of high-quality research and critical discourse. While the journal’s growing Impact Factor and readership highlight its academic reach, its value lies in supporting the ongoing advancement of oncology by promoting rigorous, timely, and clinically relevant contributions. For surgical oncologists, this presents both an opportunity and a responsibility to engage with emerging data, challenge existing paradigms, and lead innovation in cancer care. Recent contributions to *Current Oncology* have highlighted the evolving landscape of surgical oncology, emphasizing multidisciplinary, patient-centered approaches and critically examining the role of artificial intelligence (AI) within this context.

For example, the review by Guerra-Londono et al. on prehabilitation in adults undergoing cancer surgery offers a structured analysis of preoperative optimization strategies, emphasizing their impact on postoperative outcomes and long-term recovery [1]. In addition to disease-specific advances, broader metabolic factors relevant to surgical outcomes have been explored. For example, the review by Szablewski discusses the mechanistic links between insulin resistance and increased cancer risk, highlighting an important intersection between metabolic health and disease development [2].

Nardone et al. explore the integration of artificial intelligence into multidisciplinary tumor boards, highlighting differing perspectives and expectations among surgeons, medical oncologists, and radiation oncologists and emphasizing AI’s potential to enhance decision-making while underscoring the need for tailored implementation across specialties [3]. Similarly, Caglayan et al. critically assess the role of large language models in oncology, discussing their transformative potential alongside concerns about accuracy, bias, and clinical applicability and calling for cautious adoption supported by robust validation and human oversight [4].

These insights reflect a broader shift in surgical oncology, from isolated procedural expertise to an integrative, patient-centered discipline in which new technologies play a focal role. In this context, the surgical oncologist must not only master evolving oncologic principles but also navigate a landscape that demands both specialization and versatility.

As surgical strategies evolve for complex malignancies such as locally advanced pancreatic cancer, the traditional boundaries between organ-specific expertise and generalist surgical thinking are being redefined. Conversion therapy has opened new possibilities, with more patients becoming candidates for resection following induction treatment [5]. These extended resections often involve multiple organ systems, demanding not only deep oncological understanding but also technical versatility across visceral, vascular, and occasionally thoracic or urologic domains. In this setting, the generalist surgeon with high-level competence in multivisceral procedures may be uniquely positioned to deliver comprehensive surgical care. Thus, rather than a dichotomy, the future may call for a hybrid profile: a surgical oncologist with both organ-specific insight and the adaptability of a generalist.

Building on the increasing complexity of oncologic surgery—particularly in cases such as locally advanced pancreatic cancer—robotic surgery and AI are emerging as transformative tools. These technologies promise enhanced precision, real-time intraoperative decision support, and improved patient outcomes in standard oncologic cases [6,7,8,9]. However, their optimal use could go far beyond routine application in standardized cases. Implementing robotics and AI in complex cases requires surgeons who not only master the mechanics of these tools but also profoundly understand tumor biology, anatomy, and operative strategy across multiple domains. This depth of oncological and technical insight enables surgical oncologists to tailor and adapt these technologies to challenging scenarios, such as vascular resections, combined thoracoabdominal approaches, or surgery in the context of multimodal therapy. In this evolving landscape, leadership in robotic and AI-driven surgical innovation must come from those with hands-on experience in managing the full complexity of cancer surgery.

Equally essential is close integration within multidisciplinary teams, where collaborative expertise ensures that technological advances translate into meaningful oncologic outcomes. Multimodal therapy represents the gold standard in the treatment of many solid tumors, combining surgery with systemic approaches such as chemotherapy, radiotherapy, and targeted or immunotherapies to improve outcomes and reduce recurrence. [10,11,12]. Its success relies on precise sequencing and coordination, typically guided by multidisciplinary tumor boards (MDTs).

In light of these developments, the future of surgical oncology will increasingly depend on clinicians who are deeply rooted in oncologic principles and multidisciplinary practice. As innovation accelerates, there is a pressing need for thoughtful integration, where advanced techniques such as robotics and AI are employed to enhance decision-making, precision, and patient outcomes in complex scenarios. Surgical oncologists who combine subspecialty knowledge with operative breadth will be critical in shaping this transition. *Current Oncology* remains a valuable forum to document, scrutinize, and disseminate these advances, and its continued evolution will mirror the maturation of the discipline itself.
